# Supplementary material for: Acceptability and feasibility of pre-exposure prophylaxis for bacterial STIs: a systematic review
Source: PLoS One. 2025 Feb 6;20(2):e0317669. doi: 10.1371/journal.pone.0317669 (PMC11801728; doi:10.1371/journal.pone.0317669)
Supplement: S2 File — (DOCX) [file pone.0317669.s002.docx]

**Search terms by concept**

| Concept 1: Population | Concept 2a: Disease | Concept 2b: intervention | Concept 3: Outcome |
| --- | --- | --- | --- |
| patient*  service user*  client*  sex worker*  gay  bisexual  men who have sex with men  Indigenous people*  Indigenous Canadians  Australian Aboriginal and Torres Strait Islander People*  American Indian  Alaska Native  Maori People  Native Hawaiian  Other Pacific Islander  First Nation People*  health service provider*  general practitioner*  clinician*  medical practitioner*  physician*  medical doctor  health personnel  pharmacist*  infection control practitioner*  dermatologist*  urologist  gynecologist  medical staff  nurses  health care worker  health worker  medical professional  health professional  health services manager*  policymaker*  policy maker*  administrative personnel  lawmaker  ethicist*  government employee  government official* | sexually transmissible infection  sexually transmitted infection  STIs  sexually transmissible disease  sexually transmitted disease  STDs  chlamydia  gonnorrhea  gonorrhoea  syphilis  bacterial vaginosis  gardnerella vaginalis  mycoplasma genitalium  donovanosis  chancroid | antibacterial agent*  anti-bacterial agent  antibiotic*  bactericide  bacteriocide  bacteriostatic  anti-infective agent  ceftriaxone  doxycycline  azithromycin  penicillin  metronidazole  clindamycin  sitafloxacin  pristinamycin  minocycline  amoxicillin  cephalexin  cefixime  cefaxlor  AND  presumptive treatment  presumptive management  presumptive therapy  prophylactic treatment  prophylactic management  prophylactic therapy  prophylaxis  preventative treatment  preventative management  pre-exposure  preexposure  doxy prep | patient acceptance of health care  acceptability of health care  health care utilisation/utilization  health care seeking behaviour/behavior  non-acceptors of health care  treatment refusal  attitudes  treatment adherence  treatment compliance  medication adherence  patient preference  physician refusal to treat  treatment refusal  feasibility  program appropriateness  program sustainability  program effectiveness  health care evaluation  health knowledge  impact |

**Example full search strategy (PubMed)**

((((patient* OR "service user*" OR client* OR "sex worker*" OR gay OR bisexual OR "men who have sex with men" OR "Indigenous people*" OR "Indigenous Canadians" OR "Australian Aboriginal and Torres Strait Islander People*" OR "American Indian" OR "Alaska Native" OR "Maori People" OR "Native Hawaiian" OR "Other Pacific Islander" OR "First Nation People*" OR "health service provider*" OR "general practitioner*" OR clinician* OR "medical practitioner*" OR physician* OR "medical doctor" OR "health personnel" OR pharmacist* OR "infection control practitioner*" OR dermatologist* OR urologist OR gynecologist OR "medical staff" OR nurses OR "health care worker" OR "health worker" OR "medical professional" OR "health professional" OR "health services manager*" OR policymaker* OR "policy maker*" OR "administrative personnel" OR lawmaker OR ethicist* OR "government employee" OR "government official*") AND ("sexually transmissible infection" OR "sexually transmitted infection" OR STIs OR "sexually transmissible disease" OR "sexually transmitted disease" OR STDs OR chlamydia OR gonnorrhea OR gonorrhoea OR syphilis OR "bacterial vaginosis" OR "gardnerella vaginalis" OR "mycoplasma genitalium" OR donovanosis OR chancroid)) AND ("antibacterial agent*" OR "anti-bacterial agent" OR antibiotic* OR bactericide OR bacteriocide OR bacteriostatic OR "anti-infective agent" OR ceftriaxone OR doxycycline OR azithromycin OR penicillin OR metronidazole OR clindamycin OR sitafloxacin OR pristinamycin OR minocycline OR amoxicillin OR cephalexin OR cefixime OR cefaxlor)) AND ("presumptive treatment" OR "presumptive management" OR OR "presumptive therapy" OR "prophylactic treatment" OR "prophylactic management" OR "prophylactic therapy" OR prophylaxis OR "preventative treatment" OR "preventative management" OR "pre-exposure" OR preexposure OR "doxy prep")) AND ("patient acceptance of health care" OR "acceptability of health care" OR "health care utilisation" OR "health care utilization" OR "health care seeking behaviour" OR "health care seeking behavior" OR "non-acceptors of health care" OR "treatment refusal" OR attitudes OR "Treatment Adherence" OR "treatment compliance" OR "medication adherence" OR "patient preference" OR "physician refusal to treat" OR "treatment refusal" OR feasibility OR "program appropriateness" OR "program sustainability" OR "program effectiveness" OR "health care evaluation" OR "health knowledge" OR impact)
