# Supplementary material for: Acceptability and feasibility of pre-exposure prophylaxis for bacterial STIs: a systematic review
Source: PLoS One. 2025 Feb 6;20(2):e0317669. doi: 10.1371/journal.pone.0317669 (PMC11801728; doi:10.1371/journal.pone.0317669)
Supplement: S3 Table — (DOCX) [file pone.0317669.s003.docx]

Supplementary Table S1: Studies excluded in full-text review

| **No** | **Full reference** | **Reason for exclusion** |
| --- | --- | --- |
| 1 | Apers, L., Vanhamel, J., Caluwaerts, S., Platteau, T., Kenyon, C., & Florence, E. (2021). Impact of PrEP on a STI clinic in a Belgian context: a provider’s perspective. *Acta Clinica Belgica: international Journal of Clinical and Laboratory Medicine*, *76*(6), 477-481. | Study is not about bacterial STIs |
| 2 | Balkus, J. E., Carter, K. A., & McClelland, R. S. (2019). Lessons from suppressive therapy and periodic presumptive treatment for bacterial vaginosis. *Current Infectious Disease Reports*, *21*, 1-9. | Review article, no primary data collection. |
| 3 | Barbee, L., Hood, J., & Golden, M. (2018). Seattle-area MSM's attitudes towards STI diagnoses and willingness to change behavior to avoid STIs. *Sexually Transmitted Diseases, 45*(Suppl 2), S104 | This reference could not be detected anywhere in this or any other edition of this journal nor where we able to find this reference through Google searches. This reference is also not listed on any but one database. It does not appear to exist or might be an abstract for a conference that has been rescinded (given that is states ‘Suppl2 S104’ in the reference. |
| 4 | Bland, C. M., Bookstaver, P. B., Griffith, N. C., Heil, E. L., Jones, B. M., Justo, J. A., Staicu, M. L., Torney, N. P., & Wall, G. C. (2019). A practical guide for pharmacists to successfully implement penicillin allergy skin testing. *American Journal of Health-System Pharmacy, 76*(3), 136–147. | Not on pre-exposure prophylaxis (after diagnosed STI in partner/self) |
| 5 | Bohbot, J. M., Daraï, E., Bretelle, F., Brami, G., Daniel, C., & Cardot, J. M. (2018). Efficacy and safety of vaginally administered lyophilized *Lactobacillus crispatus* IP 174178 in the prevention of bacterial vaginosis recurrence. *J Gynecol Obstet Hum Reprod, 47*(2), 81–86. | Does not discuss acceptability, feasibility or knowledge translation of PrEP |
| 6 | Bolan, R. K., Beymer, M. R., Weiss, R. E., Flynn, R. P., Leibowitz, A. A., & Klausner, J. D. (2015). Doxycycline prophylaxis to reduce incident syphilis among HIV-infected men who have sex with men who continue to engage in high-risk sex: A randomized, controlled pilot study. *Sex Transm Dis, 42*(2), 98–103. | Study is not about bacterial STIs |
| 7 | Bolan, R. K., Beymer, M., Klausner, J. D., Flynn, R., & Leibowitz, A. (2013). P3. 430* Doxycycline prophylaxis for syphilis in a persistently high risk HIV infected population. *Sexually Transmitted Infections*, *89*(Suppl 1), A283-A283. | Conference presentation. Does not discuss acceptability, feasibility or knowledge translation of PrEP |
| 8 | Celum, C., & Luetkemeyer, A. F. (2021). Doxycycline for sexually transmitted infection prevention: evolving evidence and implementation perspectives. *Sexually Transmitted Diseases*, *48*(9), 620-621. | Theory paper (no primary data collection/analysis of secondary data) |
| 9 | Ellis, S. L., Tsourtos, G., Waddell, R., Woodman, R., & Miller, E. R. (2020). Changing epidemiology of gonorrhea in Adelaide, South Australia. *Sexually Transmitted Diseases, 47*(6), 402–408. | Not on pre-exposure prophylaxis (after diagnosed STI in partner/self) |
| 10 | Gao, W., Li, Z., Yan, H., Wang, D., Li, Y., Dang, S., & Qiao, X. (2012). Preventive measures against sexually transmitted infections among female sex workers in Lanzhou, China. *Scand J Infect Dis, 44*(5), 374–380. | Does not discuss acceptability, feasibility or knowledge translation of PrEP |
| 11 | Gift, T. L., Kissinger, P., Mohammed, H., Leichliter, J. S., Hogben, M., & Golden, M. R. (2011). The cost and cost-effectiveness of expedited partner therapy compared with standard partner referral for the treatment of chlamydia or gonorrhea. *Sexually Transmitted Diseases, 38*(11), 1067–1073. | Not on pre-exposure prophylaxis (after diagnosed STI in partner/self) |
| 12 | Gilbert, M., Chang, H. J., Ablona, A., Salway, T., Ogilvie, G. S., Wong, J., Haag, D., Pedersen, H. N., Bannar-Martin, S., Campeau, L., Ford, G., Worthington, C., Grace, D., & Grennan, T. (2022). Accessing needed sexual health services during the COVID-19 pandemic in British Columbia, Canada: A survey of sexual health service clients. *Sex Transm Infect, 98*(5), 360–365. | Study is not about bacterial STIs |
| 13 | Golden, M. R., & Handsfield, H. H. (2015). Preexposure prophylaxis to prevent bacterial sexually transmitted infections in men who have sex with men. *Sex Transm Dis, 42*(2), 104–106. | Does not discuss acceptability, feasibility or knowledge translation of PrEP |
| 14 | Haaland, R., Fountain, J., Dinh, C., Edwards, T., Martin, A., Omoyege, D., Conway-Washington, C., & Kelley, C. (2023). Mucosal pharmacology of doxycycline for bacterial STI prevention in men and women. *Topics in Antiviral Medicine, 31*(2), 48. | Does not discuss acceptability, feasibility or knowledge translation of PrEP |
| 15 | Hazra, A., McNulty, M. C., Pyra, M., Pagkas-Bather, J., Gutierrez, J. I., Pickett, J., ... & Klausner, J. D. (2024). Filling in the gaps: updates on doxycycline prophylaxis for bacterial sexually transmitted infections. *Clinical Infectious Diseases*, ciae062. | Theory paper (no primary data collection/analysis of secondary data) |
| 16 | Jean Cadet, M. (2018). Diagnosis, treatment, and prevention of cystitis: A case report to guide nurse practitioners. *American Nurse Today, 13*(7), 24–27. | Study is not about bacterial STIs |
| 17 | Llata, E., Braxton, J., Asbel, L., Huspeni, D., Tourdot, L., Kerani, R. P., Cohen, S., Kohn, R., Schumacher, C., Toevs, K., Torrone, E., & Kreisel, K. (2023). Presumptive and follow-up treatment associated with gonorrhea and chlamydia testing episodes in sexually transmitted disease clinics: Impact of changing treatment guidelines for gonorrhea, sexually transmitted disease surveillance network, 2015–2018. *Sex Transm Dis, 50*(1), 5–10 | Does not discuss acceptability, feasibility or knowledge translation of PrEP |
| 18 | Low, S., Varma, R., McIver, R., Vickers, T., & McNulty, A. (2020). Provider attitudes to the empiric treatment of asymptomatic contacts of gonorrhoea. *Sexual Health*, *17*(2), 155-159. | Not on pre-exposure prophylaxis (after diagnosed STI in partner/self) |
| 19 | Matser, A., Hulstein, B., de Vries, H. J. C., Hoornenborg, E., Prins, M., Davidovich, U., & Van der Loeff, M. S. (2023). What do men who have sex with men think of the use of antibiotics as pre- and post-exposure prophylaxis to prevent sexually transmitted infections? *medRxiv*. | Not peer-reviewed. A peer-reviewed version could not be found. |
| 20 | McCool-Myers, M., Smith, A. D. Y., & Kottke, M. J. (2020). Expert interviews on multilevel barriers in implementing expedited partner therapy for chlamydia. *Journal of Public Health Management and Practice*, *26*(6), 585-589. | Not on pre-exposure prophylaxis (after diagnosed STI in partner/self) |
| 21 | McCormick, D. F., Rahman, M., Zadrozny, S., Alam, A., Ashraf, L., Neilsen, G. A., ... & Hoffman, I. F. (2013). Prevention and control of sexually transmissible infections among hotel-based female sex workers in Dhaka, Bangladesh. *Sexual Health*, *10*(6), 478-486. | Not on pre-exposure prophylaxis (after diagnosed STI in partner/self) |
| 22 | Nanhoe, A. C., Visser, M., Omlo, J. J., Watzeels, A. J., van den Broek, I. V., & Götz, H. M. (2018). A pill for the partner via the chlamydia patient? Results from a mixed method study among sexual health care providers in the Netherlands. *BMC Infectious Diseases*, *18*, 1-10. | Not on pre-exposure prophylaxis (after diagnosed STI in partner/self) |
| 23 | Okah, E., Arya, V., Rogers, M., Kim, M., & Schillinger, J. A. (2017). Sentinel surveillance for expedited partner therapy prescriptions using pharmacy data, in 2 New York City neighborhoods, 2015. *Sexually Transmitted Diseases*, *44*(2), 104-108. | Not on pre-exposure prophylaxis (after diagnosed STI in partner/self) |
| 24 | Oliver, A., Rogers, M., & Schillinger, J. A. (2016). The impact of prescriptions on sex partner treatment using expedited partner therapy for Chlamydia trachomatis infection, New York City, 2014–2015. *Sexually Transmitted Diseases*, *43*(11), 673-678. | Not on pre-exposure prophylaxis (after diagnosed STI in partner/self) |
| 25 | Phillips, G., Neray, B., Janulis, P., Felt, D., Mustanski, B., & Birkett, M. (2019). Utilization and avoidance of sexual health services and providers by YMSM and transgender youth assigned male at birth in Chicago. *AIDS Care*, *31*(10), 1282-1289. | Does not discuss acceptability, feasibility or knowledge translation of PrEP |
| 26 | Pugsley, R. A., & Peterman, T. A. (2019). Presumptive and follow-up treatment for gonorrhea and chlamydia among patients attending public health department clinics in Virginia, 2016. *Sex Transm Dis, 46*(3), 199–205. | Does not discuss acceptability, feasibility or knowledge translation of PrEP |
| 27 | Reichert, E., & Grad, Y. H. (2023). Resistance and prevalence implications of doxycycline post-exposure prophylaxis for gonorrhea prevention in men who have sex with men: A modeling study. *medRxiv*. | Not on pre-exposure prophylaxis (after diagnosed STI in partner/self) |
| 28 | Sanders, E. J., Wahome, E., Okuku, H. S., Thiong'o, A. N., Smith, A. D., Duncan, S., ... & Graham, S. M. (2014). Evaluation of WHO screening algorithm for the presumptive treatment of asymptomatic rectal gonorrhoea and chlamydia infections in at-risk MSM in Kenya. *Sexually Transmitted Infections*, *90*(2), 94-99. | Does not discuss acceptability, feasibility or knowledge translation of PrEP |
| 29 | Schmidt, R., Carson, P. J., & Jansen, R. J. (2019). Resurgence of syphilis in the United States: An assessment of contributing factors. *Infectious Diseases: Research & Treatment, 12*, N.PAG. | Does not discuss acceptability, feasibility or knowledge translation of PrEP |
| 30 | Scott, H. M., & Klausner, J. D. (2016). Sexually transmitted infections and pre-exposure prophylaxis: Challenges and opportunities among men who have sex with men in the US. *AIDS Research and Therapy, 13*(1), 5 | Study is not about bacterial STIs |
| 31 | Steen, R., Chersich, M., & de Vlas, S. J. (2012). Periodic presumptive treatment of curable sexually transmitted infections among sex workers: Recent experience with implementation. *Curr Opin Infect Dis, 25*(1), 100–106. | Theory paper (no primary data collection/analysis of secondary data) |
| 32 | Steen, R., Chersich, M., Gerbase, A., Neilsen, G., Wendland, A., Ndowa, F., Akl, E. A., Lo, Y. R., & de Vlas, S. J. (2011). Periodic presumptive treatment of curable STIs among sex workers: A systematic review. *AIDS*. | Theory paper (no primary data collection/analysis of secondary data) |
| 33 | Tran, N. K., Goldstein, N. D., & Welles, S. L. (2022). Countering the rise of syphilis: A role for doxycycline post-exposure prophylaxis?. *International Journal of STD & AIDS*, *33*(1), 18-30. | Does not discuss acceptability, feasibility or knowledge translation of PrEP |
| 34 | Wahl, A., Chresten, O., & Bygvraa, D. A. (2020). An analysis on prevention interventions: Lessons learned. *International Journal of Caring Sciences, 13*(1), 207–214. | Does not discuss acceptability, feasibility or knowledge translation of PrEP |
| 35 | Wambiya, E. O. A., Atela, M., Eboreime, E., & Ibisomi, L. (2018). Factors affecting the acceptability of isoniazid preventive therapy among healthcare providers in selected HIV clinics in Nairobi County, Kenya: a qualitative study. *BMJ Open*, *8*(12), e024286. | Study is not about bacterial STIs |
| 36 | Wi, T., Ramos, E. R., Steen, R., Esguerra, T. A., Roces, M. C. R., Lim-Quizon, M. C., Neilsen, G., & Dallabetta, G. (2006). STI declines among sex workers and clients following outreach, one-time presumptive treatment, and regular screening of sex workers in the Philippines. *Sexually Transmitted Infections, 82*(5), 386–391. | Does not discuss acceptability, feasibility or knowledge translation of PrEP |
| 37 | Woodward, S. C., Tyson, H. A., & Martin, S. J. (2020). An observational study of the acceptability of patient-delivered partner therapy for management of chlamydia. *Sex Health, 17*(4), 381–383. | Not on pre-exposure prophylaxis (after diagnosed STI in partner/self) |
| 38 | Xiridou, M., Soetens, L. C., Koedijk, F. D., VAN DER Sande, M. A., & Wallinga, J. (2015). Public health measures to control the spread of antimicrobial resistance in *Neisseria gonorrhoeae* in men who have sex with men. *Epidemiol Infect, 143*(8), 1575–1584. | Not on pre-exposure prophylaxis (after diagnosed STI in partner/self) |
| 39 | Yaesoubi, R., Cohen, T., Hsu, K., Gift, T. L., Chesson, H., Salomon, J. A., & Grad, Y. H. (2020). Adaptive guidelines for the treatment of gonorrhea to increase the effective life span of antibiotics among men who have sex with men in the United States: A mathematical modeling study. *PLoS Med, 17*(4), e1003077. | Not on pre-exposure prophylaxis (after diagnosed STI in partner/self) |
| 40 | Yarwood, T. (2022). Antimicrobial resistance in gonorrhoea: what is the way forward?. *Internal Medicine Journal*, *52*(3), 354-355. | Theory paper (no primary data collection/analysis of secondary data) |
