# Supplementary material for: Acceptability and feasibility of pre-exposure prophylaxis for bacterial STIs: a systematic review
Source: PLoS One. 2025 Feb 6;20(2):e0317669. doi: 10.1371/journal.pone.0317669 (PMC11801728; doi:10.1371/journal.pone.0317669)
Supplement: S5 Table — (DOCX) [file pone.0317669.s005.docx]

S1 Table 2: Characteristics of included studies

| First Author (Year) | Country | Population | Sample size | Study Type | Outcome (summary) | Data extractors | Date of Data Extraction | Confirmation of Eligibility to be Included |
| --- | --- | --- | --- | --- | --- | --- | --- | --- |
| Arapali (2023) | Australia | MSM^1^ | 1374 | Cross-sectional | More than half (n = 732 [54.3%]) of the participants were classified as willing to use daily STI-PrEP (18.9% [n = 254]) somewhat agreed and 35.5% [n = 478] strongly agreed they would be willing to use daily STI-PrEP.  Willingness to use daily STI-PrEP was associated with participants who believed they were at higher risk of acquiring STIs and had a strong desire to avoid STIs. Those who were less willing to use daily STI-PrEP used nondaily HIV-PrEP dosing regimens, preferred to use event-driven HIV-PrEP, were university educated, and were more concerned about long-term adverse effects of HIV-PrEP  Multivariate analysis showed willingness to use daily STI-PrEP was independently associated with sex with more than 10 sexual partners in the last 6 months (aOR, 1.33; 95% CI, 1.03–1.70); methamphetamine use in the last 6 months (aOR, 1.48; 95% CI, 1.10–1.99); interest in avoiding acquiring STIs (aOR, 1.22; 95% CI, 1.11–1.35); belief that they had acquired more STIs after starting HIV-PrEP than before (aOR, 1.07; 95% CI, 1.01–1.14); willingness to take HIV-PrEP while at risk of acquiring HIV (aOR, 1.22; 95% CI, 1.04–1.44); and only using condoms at the request of a sexual partner (aOR, 1.15; 95% CI, 1.07–1.23; Table 2). In addition, those willing to use daily STI-PrEP were less likely to be university educated (aOR, 0.65; 95% CI, 0.51–0.83), use nondaily dosing regimens of HIV-PrEP (aOR, 0.56; 95% CI, 0.38–0.82), prefer event-driven HIV-PrEP (aOR, 0.69; 95% CI, 0.48–0.99), and be concerned about long-term adverse effects of HIV-PrEP (aOR, 0.90; 95% CI, 0.83–0.97; Table 2). | SW, DD | Nov 2023 | Yes |
| Fusca (2020) | Canada | MSM | 424 | Cross-sectional | Participants showed high interest in syphilis PrEP/PEP. Specifically, 60.1% expressed a willingness to use syphilis PEP, while a slightly lower proportion, 44.1%, indicated a willingness to use syphilis PrEP. This interest in syphilis prevention strategies was in the context of growing concern over STI rates in Canada, particularly syphilis, which has been on the rise since the 1990s. The study also examined participants' willingness to use HIV PrEP and PEP, with a higher acceptance rate observed: 74.0% for HIV PrEP and 75.2% for HIV PEP among HIV-negative participants.  Multivariable logistic regression analysis identified several factors associated with the willingness to use syphilis PrEP/PEP. Key predictors included a subjective assessment of STI risk, such as feeling at risk for syphilis and being very concerned about acquiring STIs, as well as objective risk factors like the number of different STIs previously diagnosed. Interestingly, prior use of HIV PrEP was strongly associated with a willingness to use syphilis PrEP, suggesting a potential overlap in the acceptance of different PrEP modalities within at-risk populations. The study underscores a substantial interest in syphilis PrEP/PEP among gbMSM, highlighting the need for further research and potential implementation of these prevention strategies. | SW, DD | Nov 2023 | Yes |
| Horn (2020) | Australia | MSM | 13 | Qualitative | Participants were interested in taking a regular antibiotic to prevent bacterial STIs, occasionally under the condition that more information was available None of the men in this study consistently used condoms and many preferred condomless anal intercourse. therefore STI-PrEP was an ‘attractive’ preventative that would provide consistent protection across multiple contexts. STI-PrEP as a way of avoiding experiences with STIs, particularly the treatments for STIs, some of which involve painful intramuscular injections  Participants indicated they would be willing to tolerate side effects or, at a minimum, trial the prophylactic regimen with some concern about the duration of side effects. Participants were also concerned about the long-term side effects of taking a regular antibiotic, e.g., the effect on normal gastrointestinal flora and antibiotic resistance  STI-PrEP was viewed by some participants as a potential catalyst for increasing conversation and transparency around STIs in the gay and bisexual community. The potential for destigmatisation was often compared with that observed with HIV-PrEP, and some participants described the potential of STI-PrEP to challenge the ‘clean’ or ‘dirty’ dichotomy in the sexually active gay and bisexual community  Daily dosing of STI-PrEP was preferred almost unanimously compared with event-driven or episodic strategies and would offer greater security for those having regular condomless anal intercourse. Even drivien were viewed as too complicated | SW, DD | Nov 2023 | Yes |
| Nath (2019) | Canada | MSM | 25 | Qualitative | Men living with HIV or with a prior diagnosis of syphilis were very interested in syphilis PrEP. While HIV-negative participants who did not have a history of syphilis expressed several concerns about taking a daily antibiotic for syphilis prevention - antibiotic resistance, unknown side effects to taking a daily antibiotic  The cost of syphilis PrEP (for both HIV-negative participants and participants living with HIV) was a potential barrier to accessing prophylaxis.  Sex-related stigma - Some participants thought that syphilis PrEP was only meant for those who were ‘very promiscuous’ and believed that they did not fall in that category: syphilis PrEP might be appropriate for ‘other’ GBM. not ‘sexually active enough’ for the drug and that it was only meant for ‘deviant(s)’.  Participants living with HIV or with a prior diagnosis of syphilis were interested in syphilis PrEP. syphilis PrEP would afford them the convenience of not having to worry about syphilis | SW, DD | Nov 2023 | Yes |
| Park (2021) | United States | MSM / HCP^2^ | 212 (MSM)  76 (HCP) | Cross-sectional | Among MSM: 67.5% of MSM responded either “probably yes” or “definitely yes” to taking doxycycline PrEP/PEP if offered by their provider. 52.4% of MSM preferred a one-time dosing of doxycycline PEP, whereas 40.1% chose a daily dosing of Doxy-PrEP.  On a scale from “not concerned at all = 1” to “very concerned = 5,” MSM had the highest level of concern for “possible drug resistance” (Likert average, 3.54) and “possible side effects” (Likert average, 3.08; (Fig. 2). “judgment from peers” was the lowest (Likert average, 1.50). Odds of agreeing of agreeing to doxycycline PrEP/PEP were lower for participants who self-identified as Asian (odds ratio [OR], 0.289; 95% confidence interval [CI], 0.098–0.850; P = 0.02) or multiracial (OR, 0.146; 95% CI, 0.043–0.496; P ≤0.01) compared with who identified as Caucasian/White. Linear-by-linear association test also revealed a trend of higher acceptability of doxycycline PrEP/PEP with increasing levels of concern regarding STIs. Those with a history of bacterial STI diagnosis within the past 12 months had 2.8 times the odds of accepting doxycycline PrEP/PEP than did those without (OR, 2.803; 95% CI, 1.218–6.448; P = 0.02), and those who are currently on HIV PrEP had 3.7 times the odds than did those who are not (OR, 3.679; 95% CI, 1.642–8.243; P ≤ 0.01).  Among HCP: less than half (43.4%) responded either “agree” or “strongly agree” when asked if they would be willing to prescribe doxycycline PrEP/PEP to their patients who are MSM - but under the hypothetical condition that the CDC has recommended the strategies, acceptability increased to 89.5%. 80.3% of healthcare providers claimed they were concerned about drug resistance regarding the prophylactic use of antibiotics | SW, DD | Nov 2023 | Yes |
| Stahlman (2015) | United States | MSM | 19 | Cohort | People were eligible to participate if they were diagnosed with early syphilis at least twice in the last five years.   15 of 19 participants said that they would use a website devoted to syphilis information and testing. They noted that a website would help high-risk MSM identify whether they were experiencing syphilis symptoms and ﬁnd a local testing or treatment venue.  Most respondents viewed an automated Web-based reminder to get tested every 3 months favourably. Among those who thought an automated reminder system was a good idea, nearly half noted that they preferred emails, whereas the other half preferred text messages.The preference for emails related to concerns that someone else such as a partner | DH, DD | Nov 2023 | Yes |
| Wilson (2013) | Australia | MSM | 4125 | Mixed methods | 52.7% (95% conﬁdence interval [CI], 50.6%–54.8%) of participants were willing to take pills every day. When asked if they would be willing to do so if it would help reduce infections in the gay community as a whole, 75.8% (95% CI, 74.0%–77.6%) said they would be likely to do so. Participants living with HIV were slightly more likely than nonpositive men to indicate being very likely to take pills every day to protect themselves from infection (33.2% vs. 25.2%, p = 0.022), but there was no difference in their likelihood to do so to reduce infections in the community.  Men in the focus group were open to and positive about the prospects of the availability of an antibiotic to reduce syphilis infections. | DH, DD | Nov 2023 | Yes |
| Zhang (2022) | China | MSM | 725 | Cross-sectional | The majority of participants, 90.6% (657), expressed their willingness to adopt chemoprophylaxis. Within this group, 542 participants were open to using chemoprophylaxis for both HIV and syphilis, while 22 showed interest solely in syphilis chemoprophylaxis. Through Pearson’s χ2 analysis, we pinpointed various factors linked to the acceptance of syphilis chemoprophylaxis, including educational attainment, sexual orientation, history of hard-drug use, syphilis testing history, prior doxycycline use, awareness of HIV chemoprophylaxis, and the perceived necessity of chemoprophylaxis for syphilis or HIV.  Further analysis using binary logistic regression, while adjusting for significant factors, showed that MSM who deemed syphilis chemoprophylaxis unnecessary (adjusted odds ratio [aOR] = 0.783, 95% confidence interval [CI] = 0.698–0.879) or who had previously used doxycycline (aOR = 0.814, 95% CI = 0.699–0.949) were significantly less inclined to accept syphilis chemoprophylaxis compared to those who had either undergone syphilis testing before or had never used doxycycline. | DH, DD |  |  |

1 Men who have sex with men; 2 Health Care Professionals
