# Supplementary material for: Acceptability and feasibility of pre-exposure prophylaxis for bacterial STIs: a systematic review
Source: PLoS One. 2025 Feb 6;20(2):e0317669. doi: 10.1371/journal.pone.0317669 (PMC11801728; doi:10.1371/journal.pone.0317669)
Supplement: Attachment — Submitted filename: Response to Reviewer Comments.docx [file pone.0317669.s006.docx]

**Response to Reviewer Comments**

**Editorial Comments**

| Please ensure that your manuscript meets PLOS ONE's style requirements, including those for file naming. | We have amended the file names to meet the style requirements. This has been a misinterpretation of the style guides. |
| --- | --- |
| Thank you for submitting the above manuscript to PLOS ONE. During our internal evaluation of the manuscript, we found significant text overlap between your submission and previous work in the [introduction, conclusion, etc.].    We would like to make you aware that copying extracts from previous publications, especially outside the methods section, word-for-word is unacceptable. In addition, the reproduction of text from published reports has implications for the copyright that may apply to the publications.    Please revise the manuscript to rephrase the duplicated text, cite your sources, and provide details as to how the current manuscript advances on previous work. Please note that further consideration is dependent on the submission of a manuscript that addresses these concerns about the overlap in text with published work. | We sought clarification on this matter via an email sent on 3 October, but unfortunately, we did not receive a response before the submission deadline. To better understand the overlap issue, we conducted a thorough review of the manuscript using iThenticate software. The results showed no concerning overlap or evidence of plagiarism.  The highlighted sections primarily consisted of:   - Commonly used phrases in our field of research (e.g., “gay and bisexual men and other men who have sex with men”), - Names of official strategies (e.g., “Global health sector strategies on HIV, viral hepatitis and sexually transmitted infections for the period 2022-2030”), - Names of relevant organisations (e.g., “the Australasian Society for HIV, Viral Hepatitis and Sexual Health Medicine”), and - Definitions used in the methodology section, which are appropriately quoted and referenced. - Additionally, other flagged instances were isolated words or standard phrasing (e.g., “a 74% reduction in syphilis”), which are routine in scientific reporting.   Given this, we are unsure of the specific areas that require revision. If this problem persists from your perspective, we would appreciate further, more specific guidance. |
| As required by our policy on Data Availability, please ensure your manuscript or supplementary information includes the following:  A numbered table of all studies identified in the literature search, including those that were excluded from the analyses.  For every excluded study, the table should list the reason(s) for exclusion.  If any of the included studies are unpublished, include a link (URL) to the primary source or detailed information about how the content can be accessed.  A table of all data extracted from the primary research sources for the systematic review and/or meta-analysis. The table must include the following information for each study:  Name of data extractors and date of data extraction  Confirmation that the study was eligible to be included in the review.  All data extracted from each study for the reported systematic review and/or meta-analysis that would be needed to replicate your analyses.  If data or supporting information were obtained from another source (e.g. correspondence with the author of the original research article), please provide the source of data and dates on which the data/information were obtained by your research group.  If applicable for your analysis, a table showing the completed risk of bias and quality/certainty assessments for each study or outcome.  Please ensure this is provided for each domain or parameter assessed. For example, if you used the Cochrane risk-of-bias tool for randomized trials, provide answers to each of the signalling questions for each study. If you used GRADE to assess certainty of evidence, provide judgements about each of the quality of evidence factor. This should be provided for each outcome.  An explanation of how missing data were handled.  This information can be included in the main text, supplementary information, or relevant data repository. Please note that providing these underlying data is a requirement for publication in this journal, and if these data are not provided your manuscript might be rejected. | Our original submission was based on the author information available on the PLOS One website under “Systematic reviews and meta-analyses” and the data availability policy, which do not provide much of the information contained in this comment. We have reviewed this comment and actioned the following:   - We added a table of articles that have been excluded at full-text review, including reason for exclusion - We have added additional information on the data extractors, date of extraction and confirmation of eligibility to the table of included articles. Please note that we were unable to confirm the exact day of the data extraction but the month. - We confirm that all relevant information was extracted from the articles and that no additional information has been received through means other than the articles themselves. - We note that the MMAT (quality assessment) has already been attached to the original submission and has been completed as per instructions provided by the original authors of the quality assessment tool. |

**Reviewer Comments**

| The authors can remove infection from the term “STI infection” since the I in STI refers to infection. | All instances have been removed/changed accordingly. |
| --- | --- |
| Please review this manuscript for grammar and sentence structure. Throughout there are errors causing incomprehension of sentences. | The manuscript has been thoroughly reviewed by the team. |
| Please define the purpose of the study clearly (give examples of the types of presumptive treatment of interest) and then use consistent language/terms throughout the manuscript. | We have changed the terminology to STI PrEP throughout for consistency and to provide clealer understanding of our work:   “Thus, the aim of this review is to systematically investigate the *extent to which existing research examines the acceptability and feasibility of population-based STI PrEP and models of care for bacterial STIs among high-risk populations and clinicians. STI PrEP, for the purpose of this systematic review, is defined as the use of antimicrobial therapy in asymptomatic individuals who are at risk of the primary acquisition of bacterial sexually transmitted infections, administered prior to a potential exposure.*”  We have also ensured that this term has been used consistently throughout the manuscript. However, there are instances that use different language, particularly in the introduction to differentiate between different strategies described in the literature. |
| When discussing the articles, please be clear. Clearly indicate the study and at minimum include the study design and population each time a study is mentioned as readers might not be able to find mention of the study earlier in the text. | This has been added throughout. |
| Please provide more details in the Methods section. I, potentially like other readers, am surprised to see that only eight studies met the inclusion criteria for this review. Since most people only read the Abstract, this will be very useful to have more details here. | We believe that much of this justified confusing results from our previously inconsistent use of terminology. Since this has been rectified, we believe that it is clearer and also explains the limited number of articles that meet the inclusion criteria. |
| The introduction is very long and contains a lot of unnecessary detail. Also, the authors do not even introduce the focus of the article, STI PrEP until page 5. All details prior to this paragraph could be significantly shortened. For example, paragraph 70-79 could be shortened to one sentence. | We agree that we might went overboard with the introduction section. The introduction section has been streamlined to focus more on STI PrEP and as a result has been shortened dramatically. |
| Potentially a typo on lines 95-96 – similar language used twice. | This has been revised. |
| Likely typo 136-139 – couldn’t understand sentence. | This has been corrected:  “A recent randomised trial conducted in the USA with MSM reported the efficacy of a daily dosing regimen of doxycycline PrEP, with a US randomised controlled trial among MSM living with HIV recording a 73% reduction in syphilis, chlamydia, and gonorrhoea incidences among the treatment group [26].” |
| I did not realize until the end of the Methods section that the focus of this review did not include STI PEP. I think one reason for this is the authors introducing so many constructs in the Introduction – I’m not sure what is Background and what is framing the purpose of the article. Another reason is potentially interchanging presumptive treatment with STI PrEP. It would be helpful for the authors to define the scope of their manuscript and review and choose one term to define this. For example, presumptive treatment needs further definition. It can mean mass treatment of a community, symptomatic treatment without a diagnosis, daily STI PrEP, etc. | We agree that the intent of the manuscript itself was not clear until readers are well into the manuscript. As mentioned prior, we have streamlined the introduction and introduced this concept in the first paragraph. The additionally added definition assists further into guiding readers through the manuscript with a much clearer understanding of topic of the review straight from the start. |
| It would be helpful for the authors to list the search terms for each concept as this will ease reproducibility. | A full search strategy has been provided in the supplementary materials. We have added a table to this that is structure more around the tables providing a easier overview of the concepts included. |
| Can the authors explain their rationale for beginning the STI presumptive treatment search to correlate with HIV PrEP guidelines? | Apologies that this has not been further clarified in the methods section. We have added a justification for this cut-off date:  “This cut-off date has been chosen to align with this important development in the field of pre-exposure prophylaxis for HIV that in the aftermath demonstrated a significant shift in approaches to treatment, sexual risk behaviours and the acceptance and update of pre-exposure prophylaxis, particularly in high-risk groups such as men who have sex with men. Furthermore, this cut-off ensures that included studies are consistent with contemporary understandings of PrEP.” |
| Line 189 - Bacterial vaginosis is not an STI. It is dysbiosis and shift of normal vaginal flora. This is not a disease state that would require presumptive treatment. | Upon further research, we agree that STI PrEP is not an appropriate treatment in this area. Given that none of the included studies have looked at bacterial vaginosis, this has no impact on the manuscript otherwise. |
| Can the authors describe what they mean by “knowledge transfer” when this is first introduced – prior to the definition in the Methods section? | We acknowledge that the term "knowledge transfer" was introduced without an explanation in the early sections of the manuscript, which may have caused confusion. We have now added a definition of this to the introduction section including its relevance to the topic. We believe that this will provide clarity to the readers right from the start. |
| Line 238 – cite the mixed methods study. | Added |
| The sentence from 245-247 is confusing as written. It was initially unclear to me that the second percentage referred to a different study. It would help to also contextualize the Park study (setting). | Agreed. We have amendend this sentence. |
| The sentence from 250-253, please state the comparator group of 44.1% for clarity. | This has been clarified. |
| Lines 258-261, how did this study measure the change in healthcare workers acceptability of STI PrEP if the Park study is a cross-sectional study as stated above. | This was measured in the same group of people based on a theoretical scenario of endorsement by the CDC; i.e., 43.3% are currently willing to prescribe STI PrEP while 89.5% would be willing to do so if it would be endorsed by the CDC. This has been clarified. |
| Lines 294-296, the authors mention a study in China, but do not complete this thought. | This has been clarified and the relevant reference has been added. |
| Line 315-318, are women more cautious or have they been excluded from major studies due to fear of pregnancy? If the authors are going to make this argument, they should tease this out a bit more. | Most of the studies had a particular focus on gay and bisexual men, which is also mirrored in the vast majority of research on HIV PrEP. As such, women were automatically excluded from these studies, regardless of being pregnant or not. Our main point is indeed, that there are very little efforts to conduct research on this in women, given that gay and bisexual men are hardly the only population at risk of STIs. We undertook a literature search and were only able to find information on pregnancy as an exclusion from earlier trials of HIV PrEP. Given the lack of information, we would like to be cautious about speculating further on this topic. |
| Line 323-325 – the authors are comparing heterosexual women and sexual minority women to “sexual minority counterparts” – this doesn’t make sense since both arms of the comparison include sexual minorities. | This was meant to read ‘sexual minority men’ and has been amended accordingly. |
| 330-334, the authors posit that risk compensation due to birth control and HIV PrEP have contributed to the rising STI rates. While this is one theory, it is controversial. Birth control has been present since the 1970’s yet STIs were not rising back then. Could the authors contextualize the idea of risk compensation and also consider other arguments for rising STIs. | We agree that the relationship between risk compensation, birth control, HIV PrEP, and rising STI rates is a complex and, at times, controversial topic. While we acknowledge that birth control has been available since the 1970s without a corresponding rise in STI rates at that time, more recent evidence suggests that evolving attitudes toward sexual risk-taking, combined with the advent of highly effective interventions like HIV PrEP and modern contraception, have played a role in risk compensation behaviours.  Risk compensation theory posits that when individuals perceive themselves as protected from one risk (e.g., pregnancy or HIV), they may be more willing to engage in riskier sexual behaviours, such as reduced condom use, which can lead to higher rates of other STIs. This is supported by recent studies, particularly among populations using HIV PrEP, where reduced condom use has been observed alongside a corresponding increase in bacterial STIs.  We also recognise that there are other factors contributing to rising STI rates, such as increased STI testing, changing sexual networks, and growing antimicrobial resistance.  To address these complexities, we have strengthened the discussion by incorporating alternative explanations while maintaining that risk compensation remains a valid and relevant theory that is supported by contemporary research. |
| 338-342 – A major reason for decreased uptake among people of lower SES is a lack of resources and structural barriers – ex: food deserts, lack of insurance, etc. | We have extended this sentence and included examples beyond those already mentioned including lack of resources. |
